# A Dimerization Site at SCR-17/18 in Factor H Clarifies a New Mechanism for Complement Regulatory Control

**DOI:** 10.3389/fimmu.2020.601895

**Published:** 2021-01-21

**Authors:** Orla M. Dunne, Xin Gao, Ruodan Nan, Jayesh Gor, Penelope J. Adamson, David L. Gordon, Martine Moulin, Michael Haertlein, V. Trevor Forsyth, Stephen J. Perkins

**Affiliations:** ^1^ Division of Biosciences, Department of Structural and Molecular Biology, University College London, London, United Kingdom; ^2^ Life Sciences Group, Institut Laue Langevin, Grenoble, France; ^3^ Division of Medicine, University College London, London, United Kingdom; ^4^ Department of Microbiology and Infectious Diseases, Flinders Medical Centre and Flinders University, Bedford Park, SA, Australia; ^5^ Faculty of Natural Sciences, Keele University, Staffordshire, United Kingdom

**Keywords:** analytical ultracentrifugation, complement factor H, inflammation, molecular modelling, X-ray scattering

## Abstract

Complement Factor H (CFH), with 20 short complement regulator (SCR) domains, regulates the alternative pathway of complement in part through the interaction of its C-terminal SCR-19 and SCR-20 domains with host cell-bound C3b and anionic oligosaccharides. In solution, CFH forms small amounts of oligomers, with one of its self-association sites being in the SCR-16/20 domains. In order to correlate CFH function with dimer formation and the occurrence of rare disease-associated variants in SCR-16/20, we identified the dimerization site in SCR-16/20. For this, we expressed, in *Pichia pastoris*, the five domains in SCR-16/20 and six fragments of this with one-three domains (SCR-19/20, SCR-18/20, SCR-17/18, SCR-16/18, SCR-17 and SCR-18). Size-exclusion chromatography suggested that SCR dimer formation occurred in several fragments. Dimer formation was clarified using analytical ultracentrifugation, where quantitative *c(s)* size distribution analyses showed that SCR-19/20 was monomeric, SCR-18/20 was slightly dimeric, SCR-16/20, SCR-16/18 and SCR-18 showed more dimer formation, and SCR-17 and SCR-17/18 were primarily dimeric with dissociation constants of ~5 µM. The combination of these results located the SCR-16/20 dimerization site at SCR-17 and SCR-18. X-ray solution scattering experiments and molecular modelling fits confirmed the dimer site to be at SCR-17/18, this dimer being a side-by-side association of the two domains. We propose that the self-association of CFH at SCR-17/18 enables higher concentrations of CFH to be achieved when SCR-19/20 are bound to host cell surfaces in order to protect these better during inflammation. Dimer formation at SCR-17/18 clarified the association of genetic variants throughout SCR-16/20 with renal disease.

## Introduction

The complement system is an enzymatic cascade in the innate immunity which acts against damaged cells or invading pathogens before they can cause infection. In the alternative pathway of complement activation, non-active complement C3 is spontaneously hydrolyzed in a tickover mechanism to C3u [also known as C3(H_2_O)], which is conformationally similar to active C3b. C3u leads to the amplification of C3 cleavage through the C3 convertase, which now hydrolyses C3 to form active C3b. C3b binds to exposed cell surfaces, targeting them for immune destruction. Complement Factor H (CFH) prevents complement-mediated host cell destruction through the interaction of its C-terminus with surface-bound C3b on anionic host cell surfaces (1, 2). Thus CFH acts as a cofactor for Factor I which cleaves C3b to inactive iC3b (3).

**Figure 1:**
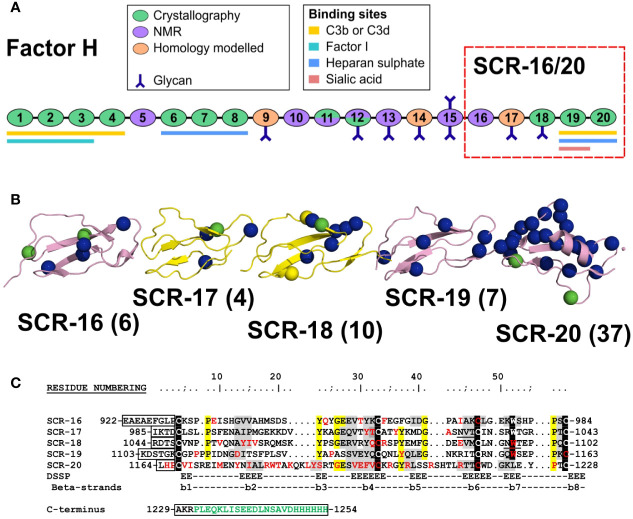
Domain structure and sequences for the Factor H C-terminal domains. **(A)** Schematic diagram of the 20 SCR domains in Factor H, showing their functional significance, knowledge of their protein structures, and their glycan chains. **(B)** Schematic view of the SCR-15/16 protein structures with their disease-associated mutations as blue spheres (aHUS), green spheres (aHUS and C3G) and yellow spheres (C3G). The number of mutations in each domain is bracketed beside the domain label. **(C)** The five domain sequences are shown, with the five conserved Trp and Cys residues highlighted in black, and other conserved residues in yellow. The two glycosylation sites are underlined. The inter-SCR linkers are boxed. Residues highlighted in grey have β-strand secondary structures. The disease-associated residues are colored in red. If expressed with a hexaHis tag, the C-terminal sequence in green will be present. The N-terminal sequence EAEAEF is the α-factor signal and the EcoRI site.

CFH is a 154 kDa glycoprotein composed of 20 short complement regulator (SCR) domains, each containing approximately 61 amino acids, and linked to each other by three to eight amino acids (**Figure 1A**) (4). There are nine N-linked glycosylation sites of which eight are occupied (5). Molecular structure determination for full length CFH is difficult due to its size, glycosylation, interdomain flexibility, and self-association. Nonetheless, high resolution structures are available for 12 SCR domain fragments of CFH solved by X-ray crystallography and for seven SCR domains solved by NMR spectroscopy (6). This leaves SCR-9, SCR-14 and SCR-17 as the only domains without high resolution structures (**Figure 1A**); however molecular models of these are available through standard homology modelling. Early electron microscopy and small angle scattering methods showed that full length CFH possesses a folded-back SCR domain structure through either its N- or C- terminals (6–8). The CFH C-terminal SCR-19 and SCR-20 domains in SCR-19/20 interact with C3b and its thioester domain C3d (9, 10). SCR-20 interacts with the cell surface through anionic interactions (11). Furthermore, CFH self-associates weakly, and that CFH forms dimers alongside higher oligomers that are directly observed as distinct peaks by analytical ultracentrifugation (AUC) (12). One of the two CFH self-association sites is localized to the five-domain fragment SCR-16/20 which exists in a monomer-dimer equilibrium, as shown using both AUC and small angle X-ray scattering (SAXS), although it was unclear from that study where the dimerization site was located in SCR-16/20 (13).

Atypical hemolytic uremic syndrome (aHUS) is a rare disease which is characterized by damage to the endothelial cells of the kidney through impaired complement regulation. It leads to end-stage renal failure and is often fatal (14). aHUS has been strongly associated with mutations in CFH (15–17). CFH-associated genetic variants cause loss of function which impairs the protection of the endothelial surfaces of the kidney, and causes complement activation on these surfaces (18). The most recent survey of CFH variants reported that there were 190 disease-associated variants in CFH (17, 19). Of these, 83 were located in the five C-terminal SCR domains of CFH. The web database (https://www.complement-db.org/) currently indicates six variants in SCR-16, four in SCR-17, ten in SCR-18, seven in SCR-19 and 37 in SCR-20 (**Figures 1B, C**). The majority of these variants are located in SCR-20 which has binding sites for C3b, C3d and anionic surfaces, demonstrating that these variants will directly perturb the ability of CFH to recognize and protect host cells. A further group of CFH variants involves 29 of the 40 disulphide bridges in CFH in which a single Cys residue is replaced, meaning that disease would be caused by protein misfolding of the SCR domain in question and the destabilisation of the CFH protein structure (19). Other complement-associated renal diseases include C3 glomerulopathy (C3G) (17).

In order to identify the CFH self-association site in SCR-16/20 and to clarify the involvement of the aHUS-associated variants in SCR-16/20 on the protein structure, we expressed seven recombinant fragments of these five C-terminal SCR domains. Using a combination of size exclusion chromatography, AUC and SAXS in both 137mM NaCl (physiological salt) and 50 mM NaCl (low salt) buffers, we identified the C-terminal dimer site in CFH to be within the double-domain SCR-17/18 region. From our AUC and SAXS results we propose that the dimer is formed by a side-by-side association of the SCR-17/18 domains, and confirmed this by recourse to recently-modelled solution structures for full-length CFH (6). We discuss the implications of our self-association results for CFH function and how genetic variants may compromise the function of CFH.

## Materials and Methods

### Expression and Purification of the CFH Fragments

In order to locate the self-association site in the SCR-16/20 region of CFH, between one to five domains in the SCR-19/20, SCR-16/20, SCR-18/20, SCR-16/18H, SCR-17/18H, SCR-17H, and SCR-18H constructs were expressed and purified for this study, where the suffix H indicated the presence of a His tag (13). The N- and C-terminal sequences of the expressed SCR domains depended on the fragment. For all seven SCR fragments, the N-termini contain the sequence EAEAF corresponding to the α-factor secretion signal and the EcoRI restriction enzyme site. The SCR-18H, SCR-17H, SCR-17/18H, and SCR-16/18H C-termini contain the first four amino acids of the next linker region followed by the myc tag and His tag sequences ALEQKLISEEDLNSAVDHHHHHH (**Figure 1C**). The SCR-16/18 and SCR-16/20 fragments contained the last three residues while the SCR-19/20 fragment contained the last four residues of the linker at its N-terminal. The SCR domains were cloned into the *Pichia pastoris* expression plasmid pPICZαA and transformed into wild-type X33 cells. Expression was carried out according to Invitrogen guidelines. Briefly, transformants were selected using zeocin given that pPICZαA encodes a zeocin resistant gene. Cell growth was maintained in media containing 2% glycerol for four days. Recombinant protein expression was induced using 0.5% methanol and was maintained every 24 h for four days. Cells were removed by centrifugation and the supernatant containing the secreted SCR domains were concentrated using a 5 kDa molecular weight cutoff membrane.

**Figure 2:**
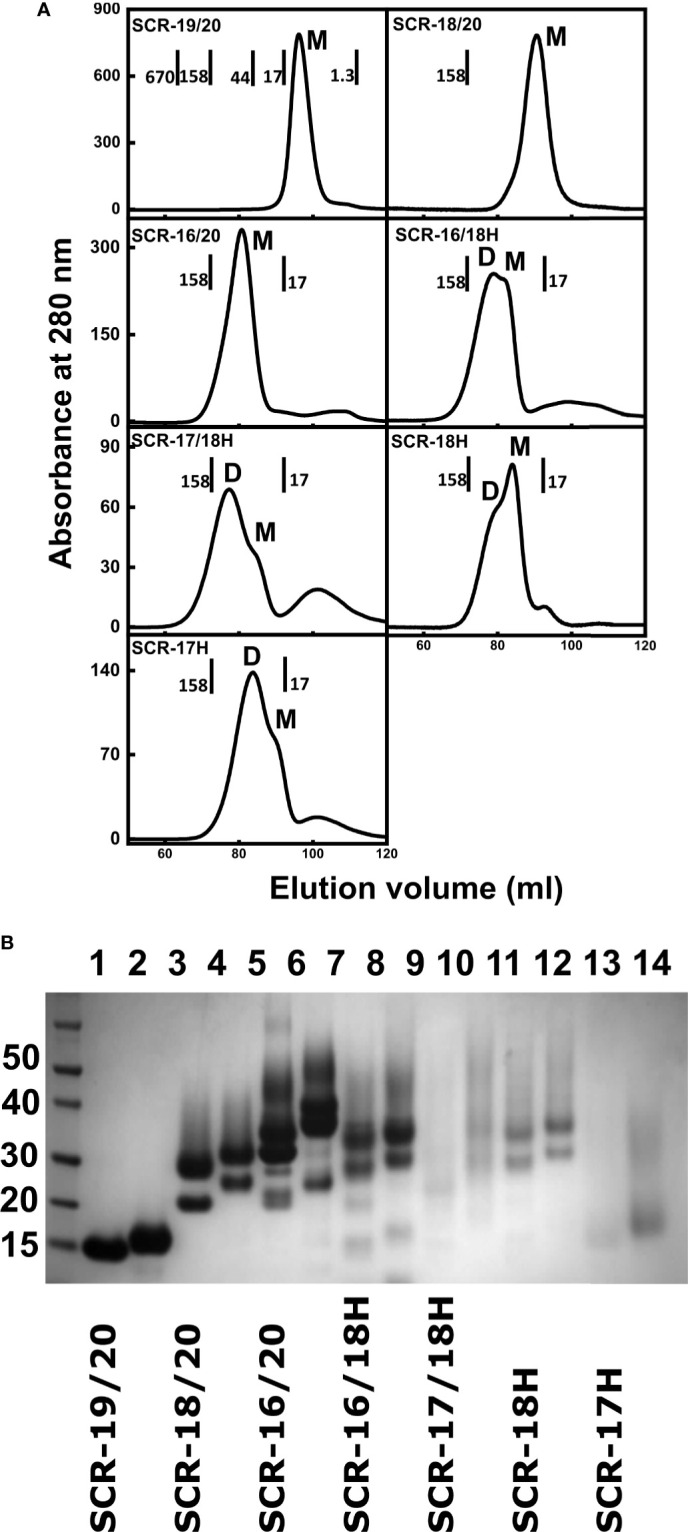
Protein purifications for seven C-terminal domains of Factor H. **(A)** Size exclusion chromatography elution profiles of each of the seven SCR fragments. The molecular mass of the calibration standards are shown in full for SCR-19/20 and in part for the others. The assignment of the peaks as monomer or dimer is denoted by M and D respectively. **(B)** SDS-PAGE analysis of each of the seven SCR fragments. Lanes 1 and 2, SCR-19/20 non-reduced and reduced respectively; lanes 3 and 4, SCR-18/20 non-reduced and reduced; lanes 5 and 6, SCR-16/20 non-reduced and reduced; lanes 7 and 8, SCR-16/18H non-reduced and reduced; lanes 9 and 10, SCR-17/18H non-reduced and reduced; lanes 11 and 12, SCR-18H non-reduced and reduced; lanes 13 and 14, SCR-17H non-reduced; and reduced. Molecular weight standards in kDa are shown to the left.

Fragments with the C-terminal hexa-histidine tags were purified using a 5 ml HiTrap Nickel column (GE Healthcare). The supernatant was dialyzed against 50 mM NaH_2_PO_4_, 300 mM NaCl, 10 mM imidazole, pH 8.0 (wash buffer), and loaded onto the column using an AKTA purifier system (GE Healthcare) which had been equilibrated with wash buffer. The column was washed with five column volumes of wash buffer to remove any non-specifically bound protein. Protein was eluted using 50 mM NaH_2_PO_4_, 300 mM NaCl, 250 mM imidazole, pH 8.0. For the non-His-tagged SCR domains, ion exchange chromatography was used. SCR-19/20, SCR-16/20 and SCR-18/20 have theoretical isoelectric points of 9.05, 8.04 and 7.69 respectively, thus cation exchange chromatography was used. The supernatant was dialyzed against 50 mM Tris-HCl pH 7.4, 25 mM NaCl, 1 mM EDTA and loaded onto a SP FF column (GE Healthcare) which had been pre-equilibrated with the same buffer. After loading, the column was washed with five column volumes of buffer. Protein was eluted using a salt gradient up to 1 M NaCl. In all seven purifications, protein elution was monitored using the absorbance at 280 nm. Fractions were pooled and concentrated using Amicon ultra centrifugal filters with a molecular weight cutoff membrane of 10 kDa or 3 kDa depending on the SCR domain. Size exclusion chromatography removed any remaining impurities and aggregation (**Figure 2A**). Protein samples were injected onto a Superdex 75 (GE Healthcare) column which had been equilibrated with 10 mM Hepes, 137 mM NaCl, pH 7.4. Molecular weight standards were from BioRad (BioRad Gel Filtration Standard, Hertfordshire, UK). SDS-PAGE monitored sample purity (**Figure 2B**).

### Analytical Ultracentrifugation of the CFH Fragments

Analytical ultracentrifugation (AUC) data were collected for each SCR fragment in two buffers, namely 10 mM Hepes pH 7.4, 137 mM NaCl and 10 mM Hepes pH 7.4, 50 mM NaCl. Experiments were carried out in two sector cells (buffer and sample) with column heights of 12 mm. Data were collected in a concentration series between 0.2 – 3 mg/ml for each fragment. Sedimentation velocity experiments were carried out at 20 °C using an AnTi50 rotor at 50,000 rpm in a Beckman-Coulter Proteome XL-I analytical ultracentrifuge. Interference and absorbance optics at 280 nm were used for detection depending on concentrations, the absorbance data being saturated at higher concentrations. Size distribution *c(s)* analyses of the sedimentation boundaries were fitted using SEDFIT according to the Lamm equation (version 14.6) to give the sedimentation coefficients *s* which were corrected to standard *s_20,w_* values to allow for the density of water and 20°C (20, 21). Totals of 80-100 boundaries were used with the frictional ratio (f*/f_0_*), meniscus and baseline all floated in the final analyses. The *c(s)* plots were converted to molar mass distribution *c(M)* in order to assess the molecular mass of each sedimenting species.

### Small Angle X-Ray Scattering of the CFH Fragments

Small angle X-ray scattering (SAXS) experiments were performed on each of the SCR domains in both 10 mM Hepes, 137 mM NaCl, pH 7.4, and 10 mM Hepes, 50 mM NaCl, pH 7.4 buffers between concentrations of 0.5–2 mg/ml. Data were collected on the bioSAXS beamline BM29 at the European Synchrotron Radiation Facility, Grenoble, France (22). The X-ray wavelength was 0.09919 nm. All experiments were carried out at 20°C. An automated capillary flow sample changer was used on BM29 in which the buffer backgrounds were measured before and after each protein sample (23). Sample volumes of 50 μl were used, collecting 10 frames at a rate of one frame per second. Frames that showed no radiation damage or aggregation were averaged, and the averaged buffer frames were subtracted from the protein scattering curves. EDNA software provided automatic data processing in which the intensities *I(Q)* were automatically scaled by concentration (24). The Biosaxs Customized Beamline Environment (BsxCuBE) software was used for control of the automatic sample changer, and the sample settings were loaded from the Information System for Protein Crystallography Beamlines database (ISPyB) (22, 25).

Guinier analyses at low *Q* (where *Q* = 4π sin θ/*λ*; 2*θ* is the scattering angle and *λ* is the wavelength) were then performed according to the Guinier equation (26).

ln I(Q)=ln I(0)-RGQ22/3

Initial data subtraction and Guinier analyses were carried out using the software Primus (27). The radius of gyration *R_G_* was calculated, which monitors the overall elongation of the protein in a given solute-solvent contrast if the internal inhomogeneity of scattering densities within the glycoprotein has no effect. The *R_G_* value was calculated from the linear portion of the Guinier plot (ln *I(Q)* v *Q^2^*) within an upper *Q*.*R_G_* limit of 1.5, together with the forward scattering intensity at zero angle *I(0)*. The program GNOM was used to transform the scattering curves in reciprocal space (*I(Q)*) into real space *via* an indirect Fourier transform to give the distance distribution *P(r)* function (28):

$$P(r)=\frac{1}{2\pi^{2}}\int_{0}^{\infty }I\left(Q\right)Qr\sin\left(Qr\right)dQ$$

The *P(r)* curve corresponds to the distribution of distances *r* between volume elements in the molecule. The *P(r)* curve yields the *R_G_* value in real space together with *L*, the maximum dimension of the molecule, and *M*, the most frequently observed interatomic distance in the molecule.

### Molecular Modelling of the Seven CFH Fragments

The most recent scattering modelling of the 20 SCR domains in CFH used a combination of MODELLER v9.14 and monomer Monte Carlo (SASSIE-web) (91) to build a starting CFH model from previously-known NMR and crystal structures for 17 SCR domains and three SCR homology models for SCR-9, SCR-14 and SCR-17 (6, 29, 30). Eight biantennary disialylated glycans were added to this CFH model (5). In four Monte Carlo simulations based on conformationally varying the inter-SCR linkers, 510,000 full-length CFH models were created, of which many were discarded for reason of steric clashes between the SCR domains to result in a library of 29,715 physically-realistic CFH models for SAXS curve fitting (6). A theoretical scattering curve was generated from each model for comparison with the experimental CFH scattering curve using the *R-*factor (31):

(1) $$R=\sum_{Q_{i}}\frac{\left|(I_{exp}(Q_{i})-I_{model}(Q_{i}))\right|}{\left|I_{exp}(Q_{i})\right|}$$

where *Q_i_* is the *Q* value of the *i*-th data point, *I*_exp_(*Q_i_*) is the experimental scattering intensity and *I*_model_(*Q_i_*) is the theoretical modelled scattering intensity. The *R*-factor vs *R_G_* graphs for 29,715 CFH models were filtered on both the *R_G_* value and *R-*factor. The best-fit 100 models were identified by ranking the filtered models by their *R-*factors. The Tyr402His polymorphism had no effect on the curve fits, leading to an *R-*factor difference of only 0.0003%, thus only the Tyr402 CFH models were used in the present study. These best-fit models are available from the **Supplementary Materials** of our earlier study (6); they are not available in the small angle scattering biological data bank (SASBDB) because this data bank is not suited to the deposition of atomistic scattering models.

In order to evaluate whether the seven CFH fragments of this study could be fitted to monomer models for their structures, each of the 100 best fit CFH Tyr402 models were edited to generate their seven fragments. Those for SCR-19/20, SCR-18/20 and SCR-16/20 were unchanged from those found in the full-length CFH models. Those for SCR-16/18H, SCR-17/18H, SCR-18H, and SCR-17H were modified by the addition of the C-terminal Histag sequence ALEQKLISEEDLNSAVDHHHHHH to the SCR models edited from the full-length CFH models (**Figure 1C**). This additional structure was added to each SCR fragment using MODELLER version 9.14. Because MODELLER does not handle glycans, the two biantennary disialylated glycan chains were reinstated on SCR-17 and SCR-18 by superimpositions using PyMol. CHARMM-GUI software was used to generate the CHARMM force field and PSF inputs for energy minimization in SASSIE-web (6). Once the two glycans were added to the SCR model and accepted by GlycanReader, bash scripts were used to finalize the nomenclature and numbering of the glycan and protein atoms in order to match the experimental protein.

For the AUC modelling, the theoretical *s°_20,w_* values for the seven FH fragments were calculated directly from the atomic coordinates with the default value of 0.31 nm for the atomic element radius for all atoms to represent the hydration shell by using the HYDROPRO shell modelling program (32, 33).

The sequence alignment of CFH SCR-17/18 with the SCR-1/2 domains of complement Factor H related-1 protein (FHR1) was carried out for the Uniprot KB sequences using the EMBOSS water sequence pairwise alignment tool (34). The SCR-17/18 domains were structurally aligned with the FHR1 SCR-1/2 domains using PyMol. This used the homology model for SCR-17 from the solution structure of CFH SCR-16/20 (13), SCR-18 from the crystal structure of CFH SCR-18/20 (35) and the crystal structure of the FHR1 SCR-1/2 dimer (PDB code: 3ZD2) (36). Alignment was carried out using the core residues of the β4 strand of each SCR domain, where SCR-17 was aligned with SCR-1, and SCR-18 was aligned with SCR-2.

## Results

### Purification of the Seven SCR Fragments of CFH

The non-tagged SCR-19/20, SCR-18/20 and SCR-16/20 fragments were successfully purified from the *P. pastoris* growth media supernatant by cation exchange chromatography (13). Size exclusion chromatography, which separates molecules based on their size and shape, was used as the final purification step. Molecular weight standards were used to estimate the molecular weight, and therefore oligomeric state, of each of the SCR fragments. Elution was monitored by absorbance at 280 nm (**Figure 2A**). SCR-19/20 eluted as a single symmetrical peak with an apparent mass of 10 kDa, which is comparable to 14.7 kDa expected for the monomer (M). Both SCR-18/20 and SCR-16/20 eluted with a single broad peak with a small shoulder peak on the left. SCR-18/20 showed an estimated mass of 77 kDa, and SCR-16/20 showed a mass of 71 kDa, both of which were much larger than the expected masses of 24 kDa and 38 kDa respectively. The discrepancies between the observed and expected molecular masses were attributed to the elongated shapes of the three fragments, in distinction to the molecular weight standards used for the column calibrations which were a set of globular proteins of compact shapes.

Four additional fragments containing one-to-three domains and His-tagged C-termini, namely SCR-16/18H, SCR-17/18H, SCR-17H, and SCR-18H, were likewise purified from the yeast supernatant using nickel affinity chromatography. SCR-16/18H, SCR-17/18H, SCR-18H, and SCR-17H eluted with two overlapping peaks that were assigned to dimer (D) and monomer (M) (**Figure 2A**). SCR-16/18H, SCR-17/18H, and SCR-17H showed more dimer than monomer, while SCR-18H showed more monomer. For SCR-16/18H, even though the predicted mass from the sequence was 29 kDa, peaks D and M showed masses of 141 kDa and 120 kDa respectively. For SCR-17/18H, even though the predicted mass from the sequence was 22 kDa, peaks D and M showed masses of 102 kDa and 84 kDa respectively. SCR-17H with a predicted mass of 10 kDa showed apparent molecular masses of 48 kDa and 23 kDa for peaks D and M respectively. SCR-18H with a predicted mass of 11 kDa showed apparent molecular masses of 79 kDa and 48 kDa for peaks D and M respectively. The discrepancies between the observed and expected molecular masses in the latter cases were attributed to the presence of both glycan chains and extended His-tag structures.

Protein purities were assessed by SDS-PAGE (**Figure 2B**). As SCR-17 and SCR-18 contained N-linked glycan chains, six of the SCR fragments (**Figure 1B**) showed streaking on the gel which is characteristic of glycosylated proteins, and seen previously for SCR-16/20 (13). SDS does not bind sufficiently to glycan chains, resulting in a non-uniform net charge in SDS-PAGE. As expected, only SCR-19/20 showed one band. Multiple bands were observed for the glycosylated fragments and attributed to variations in the glycosylation pattern which is often observed for glycoproteins expressed in *P. pastoris* (37). The purity and consequently the identity of each of the constructs used in this study was confirmed by Western blots using an anti-FH polyclonal goat antibody. With the exception of SCR-18, all bands that were present on the SDS-PAGE gel were confirmed to be FH. For SCR-18, MALDI-TOF mass spectroscopy analysis was carried out to confirm its mass as 11,180 Da, in agreement with the sequence. Mass variations of ± 700 Da were observed corresponding to the differential glycosylation pattern in SCR-18.

### AUC of the Seven CFH Fragments in 137 mM NaCl

**Figure 3:**
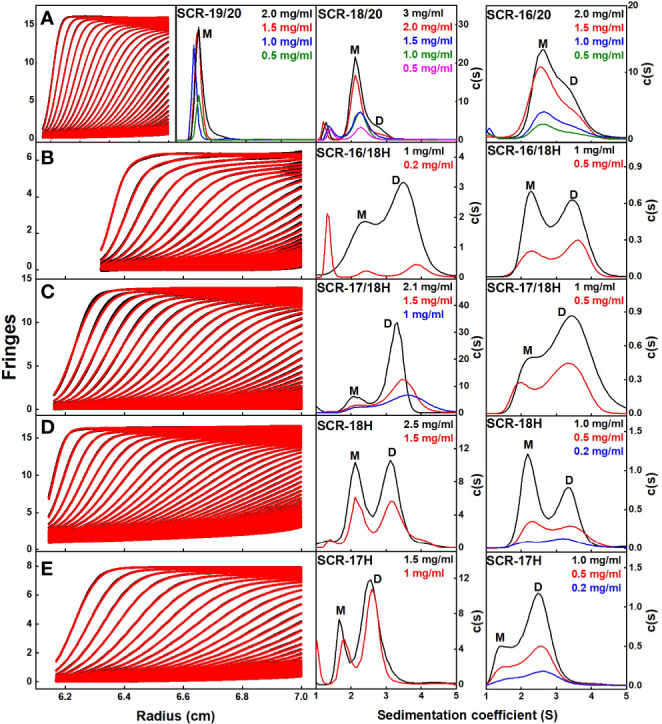
Sedimentation velocity *c(s)* distribution analyses of the C-terminal SCR fragments. In the left panels, 60–80 scans and boundary fits are shown using interference optics. Only every seventh to tenth scan is shown. In the right panels, the resulting *c(s)* distributions are shown, in which the peaks for the observed *s* values of the monomer, dimer and tetramer are denoted by M, D, and T respectively. The concentrations are shown in each panel, highlighted according to their colors in the *c(s)* distributions. **(A)** For SCR-19/20, the *c(s)* analyses showed a single monomeric peak. For SCR-18/20, the *c(s)* analyses showed mostly monomer and some dimer. For SCR-16/20, the *c(s)* analyses showed two partly resolved peaks corresponding to monomer and dimer. **(B)** For SCR-16/18H, the *c(s)* distribution on the left shows interference optics while the right shows absorbance optics. The two peaks M and D correspond to monomeric and dimeric SCR-16/18H. **(C)** For SCR-17/18H, the two peaks M and D correspond to monomer and dimer. **(D)** For SCR-18H, the two peaks M and D correspond to monomer and dimer. **(E)** For SCR-17H, the two peaks M and D correspond to monomer and dimer.

AUC quantitatively separates macromolecules according to their size and shape, this method being superior to the qualitative estimates from size-exclusion chromatography (38). Different molecular species within a sample are detected from the peaks in the size distribution *c(s)* analyses (**Figure 3**, right) that are calculated from the sedimentation boundaries (**Figure 3**, left). The sedimentation coefficient *s_20_,_w_* at 20°C and corrected for the density of water gives the frictional ratio *f/f_o_*. This measures the protein shape, with a compact globular protein typically having a *f/f_o_* ratio of 1.2 where *f* is the observed frictional coefficient and *f_0_* is the frictional coefficient for a spherical protein with the same mass. The *f/f_o_*ratio will indicate the degree of elongation upon protein dimerization. The relative percentages of monomer and dimer in the sample are calculated from peak integrations in the *c(s)* analyses. These integrations give the dissociation constant *K_D_* for dimer formation. Conversion of the *c(s)* peaks to the corresponding mass distributions *c(M)* gives the molecular mass for each species present.

Interference optics were used for the three non-His tagged fragments SCR-19/20, SCR-18/20 and SCR-16/20, where good boundary fits were obtained in all cases (**Figure 3A**). For this work, buffers with 137 mM NaCl were used that correspond closely to the ionic strength of blood plasma.

**Figure 4:**
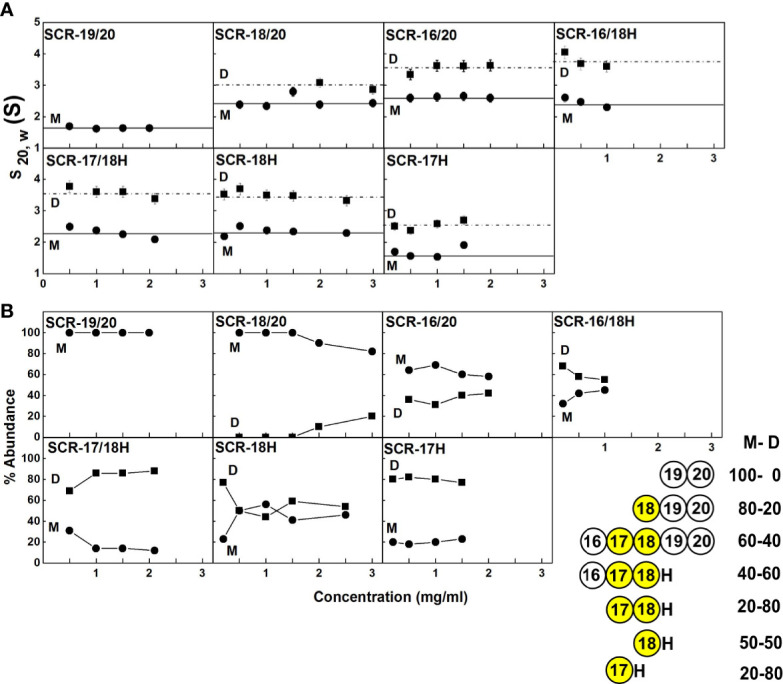
Concentration-dependence of the sedimentation data for each of the SCR fragments. **(A)** The sedimentation coefficients *s_20,w_* corrected for buffer density and temperature for the monomer (M) is denoted by filled circles, and for the dimer (D) with filled squares. Statistical error bars are shown where visible. **(B)** The relative percentage of monomer and dimer present in the *c(s)* analyses. The inset to the right summarizes the relative percentages of monomer (M) and dimer (D) from the AUC analyses. SCR-17 and SCR-18 are highlighted in yellow as the dimer site.

(i) For SCR-19/20, only one peak was visible in the *c(s)* plot for four concentrations, indicating that only monomer was present with an *s_20_,_w_* value of 1.6 S (**Figure 4**). The *c(M)* analysis gave a molecular mass of 15–18 kDa, which was in good agreement with the sequence-calculated mass of 15 kDa. The *f/f_o_* ratio for SCR-19/20 was 1.1 showing that it had a relatively compact shape (**Table 1**).

**Table 1:** AUC and SAXS parameters for the seven SCR fragments in 137 mM and 50 mM NaCl.

Domains	$s_{20,w}^{0}$ (S)	$s_{20,w}^{0}$ (S)	*f/f_0_*	*f/f_0_*	R_G_ (nm)^a^	*R_G_/R_O_*	R_G_ (nm)^a^	*R_G_/R_O_*	L (nm)	L (nm)
	137 mM	50 mM	137 mM	50 mM	137 mM	137 mM	50 mM	50 mM	137 mM	50 mM
SCR-19/20 monomer	1.64	1.8	1.1	1.04	2.4 ± 0.09	1.7	2.2 ± 0.1	1.6	8.2 ± 0.4	8.2 ± 0.4
SCR-18/20 monomer	2.4	2.4	1.1	1.1	3.2 ± 0.06	2	3.4 ± 0.1	2	12.2 ± 0.6	11.7 ± 0.6
SCR-18/20 dimer	2.9	2.9	1.6	1.6	3.62 ± 0.03	2.2	3.6 ± 0.03	2.2	12.7 ± 0.3	12.3 ± 0.6
SCR-16/20 monomer	2.59	3.1	1.5	1.3	4.7 ± 0.06	2.4	4.8 ± 0.1	2.5	16.8 ± 0.8	17.5 ± 0.8
SCR-16/20 dimer	3.63	4.2	1.7	1.5	6 ± 0.1	3.1	6.1 ± 0.1	3.2	21 ± 1	24 ± 0.2
SCR-16/18H monomer	2.47	2.2	1.3	1.5	4.7 ± 0.04	2.7	4.4 ± 0.1	2.5	17 ± 0.8	15.5 ± 0.8
SCR-16/18H dimer	3.68	3.5	1.4	1.5	4.7 ± 0.06	2.7	4.4 ± 0.01	2.5	17 ± 0.5	15.9 ± 0.8
SCR-17/18H monomer	2.49	2.2	1.3	1.3	2.7 ± 0.2	1.7	2.7 ± 0.1	1.4	9 ± 0.5	9.7 ± 0.5
SCR-17/18H dimer	3.77	3.8	1.3	1.3	2.8 ± 0.01	1.7	2.8 ± 0.01	1.4	10.2 ± 0.5	9.7 ± 0.2
SCR-18H monomer	2.51	2.32	1.2	1.3	2.2 ± 0.03	1.4	2.2 ± 0.03	1.5	8 ± 0.4	7.9 ± 0.4
SCR-18H dimer	3.49	3.69	1.3	1.3	2.2 ± 0.01	1.4	2.2 ± 0.02	1.5	8 ± 0.1	7.9 ± 0.3
SCR-17H monomer	1.56	1.82	1.2	1.7	3.5 ± 0.03	2.2	3.2 ± 0.01	2	12 ± 0.6	11.9 ± 0.6
SCR-17H dimer	2.37	2.86	1.6	1.6	3.4 ± 0.01	2.2	3.2 ± 0.1	2	12 ± 0.1	11 ± 0.6

a For the SAXS results, the monomer is taken as the value recorded at the lowest concentration (i.e. lowest dimer percentage) and the dimer is taken as the highest concentration (i.e. the highest dimer percentage).

(ii) SCR-18/20 showed one peak in the *c(s)* plot with an *s_20_,_w_* value of 2.4 S corresponding to the monomeric protein. At higher concentrations of 2–3 mg/ml, a small shoulder peak was evident with an *s*_20,w_ of 3.1 S which was attributed to a low amount of dimer formation (**Figure 3A**). Integration showed that this shoulder peak accounted for 10–18% of the sample for 2–3 mg/ml (**Figure 4B**). From this, the dissociation constant *K_D_* for the SCR-18/20 dimer was estimated to be 590 ± 150 µM. The molecular mass for the monomer was determined to be 26–34 kDa, in accord with the sequence-calculated monomer mass of 24 kDa, and 58 kDa for the shoulder peak to confirm that this was dimer. SCR-18/20 showed a *f/f_0_* ratio of 1.1 for the monomer and 1.6 for the dimer peak, showing that the dimer was more elongated than the monomer (**Table 1**).

(iii) The *c(s)* curve for SCR-16/20 showed the presence of monomer and dimer with two partially merged peaks corresponding to a monomer-dimer equilibrium, with the monomer *s_20_,_w_* value at 2.6 S and the dimer *s_20_,_w_* value at 3.6 S (**Figure 3A**), as reported previously (13). The 60–40% ratio of monomer-dimer did not significantly change with concentration (**Figure 4B**), and resulted in a *K_D_* value of 31 ± 14 µM for dimer formation. The molecular masses were determined to be 41–48 kDa for the monomer and 65–66 kDa for dimeric SCR-16/20, as expected from the sequence-calculated monomer mass of 38 kDa. The *f/f_0_* ratio was 1.5 for the monomer and 1.7 for the dimer, indicating that the dimer was slightly more elongated than the monomeric protein (**Table 1**).

In order to locate the dimerization site more precisely, four His-tagged SCR fragments were available, based on SCR-16, SCR-17, and SCR-18, and denoted by H suffixes. AUC data for these were based on both interference and absorbance optics for which again good boundary fits were obtained (**Figures 3B–E**):

(iv) For SCR-16/18H, the *c(s)* plots showed well-resolved monomer and dimer peaks, even at low concentrations of 0.2 mg/ml (**Figure 3B**). Interference optics gave *s_20_,_w_* values of 2.6 S and 2.3 S for the monomer and 4.0 S and 3.6 S for the dimer. Absorbance optics gave *s_20_,_w_* values of 2.5 S and 3.6 S for the monomer and dimer respectively. The *c(M)* analyses gave 21–32 kDa for the monomer and 40–55 kDa for the dimer, in good accord with the sequence-calculated monomer mass of 29 kDa. Integration showed that SCR-16/18H was 40% monomer and 60% dimer (**Figure 4B**), giving a *K_D_* value of 6 ± 5 µM for dimer formation. The *f/f_o_* ratio was 1.3 for the monomer and 1.4 for the dimer, indicating that the protein became slightly more elongated upon dimer formation (**Table 1**).

(v) For SCR-17/18H, the interference and absorbance data showed monomer and dimer peaks (**Figure 3C**). The *s_20_,_w_* values were 2.1 S to 2.5 S for the monomer and 3.4 S to 3.6 S for the dimer (**Figure 4A**). The experimental molecular masses were 21–24 kDa for the monomer and 44–49 kDa for the dimer, in good agreement with a sequence-calculated monomer mass of 22 kDa. SCR-17/18H existed as 80% dimer (**Figure 4B**), giving a *K_D_* for SCR-17/18H dimer formation of 3 ± 1 µM. The *f/f_0_* ratio was 1.3 for both the monomer and dimer, showing that both were relatively compact in their structures.

(vi) SCR-18H also showed two peaks in the *c(s)* distribution (**Figure 3D**). The first peak showed *s_20,w_* values of 2.2–2.5 S and the second peak showed *s_20,w_* values of 3.5–4.0 S. The two peaks were each approximately 50% in size (**Figure 4B**), giving a *K_D_* value for SCR-18H dimer formation of 37 ± 27 µM. The *c(M)* analyses gave molecular masses of 20–31 kDa for the first peak and 37–57 kDa for the second peak. Both values were double those expected from the sequence-calculated mass of 11 kDa for the monomer, thus it was not clear if the two peaks corresponded to monomer-dimer or dimer-tetramer. It is possible that the relatively large glycan chain on SCR-18H may affect the sedimentation results. Nonetheless the *f/f_0_* ratio was calculated to be similar at 1.2 for the first peak and 1.3 for the second peak assuming that these corresponded to monomer and dimer, showing that both were relatively compact in their structures.

(vii) For SCR-17H, two peaks were also evident in the *c(s)* distribution (**Figure 3E**). The first peak showed *s_20,w_*values of 1.5–1.9 S and the second peak showed *s_20,w_*values of 2.4–2.7 S. SCR-17H exists as 80% dimer (**Figure 4B**), from which the *K_D_* value for dimer formation was 5 ± 4 µM. The *c(M)* analyses gave molecular masses of 17–23 kDa for the first peak and 30–41 kDa for the second peak. As found with SCR-18H, both values were double those expected from the sequence-calculated mass of 10 kDa for the monomer, thus it was not clear if the two peaks corresponded to monomer-dimer or dimer–tetramer. Nonetheless the *s_20,w_*values were in the expected range for monomeric and dimeric SCR-17H. The *f/f_0_* ratios were calculated for monomeric and dimeric SCR-17H to be 1.14 for the monomer and 1.6 for the dimer, indicating some elongation upon dimer formation.

With the exception of SCR-19/20 which was monomeric, the other six SCR fragments each showed two distinct *c(s)* peaks corresponding to monomer and dimer. The strongest dimerization with *K_D_* values in the range of 3-6 µM was observed for the three smaller fragments when SCR-17H was present (inset, **Figure 4B**), thus it was confirmed that SCR-17H comprised the main C-terminal CFH dimer site. SCR-18H alone also showed self-dimerization. The three larger fragments showed weaker dimer formation with *K_D_* values of 31 µM, 37 µM and 590 µM.

### AUC of the Seven CFH Fragments in 50 mM NaCl

The same AUC analysis was carried out on each of the seven SCR fragments, but in low salt buffers containing 50 mM NaCl in order to act as a control for the above analyses that used 137 mM NaCl buffers. Low salt buffer will promote stronger interactions between charged groups if present. Interference and absorbance optics were used for all the SCR fragments except for SCR-16/20 when only interference optics were used (data not shown) (39).

(i) The *c(s)* distribution for SCR-19/20 again showed only one peak for monomer with an *s_20,w_* value of 1.8 S and 1.7 S between 0.5–2.0 mg/ml (**Table 1**).
(ii) The *c(s)* distribution for SCR-18/20 also again showed a major monomer peak in the *c(s)* distribution with an *s_20,w_* value of 2.2–2.3 S, together with a small shoulder peak at concentrations above 1 mg/ml with a *s_20,w_*value of 2.9 S. Because the monomer accounted for 80% of the protein, the *K_D_* value for dimer formation was estimated at 180 ± 130 µM (**Table 1**).
(iii) SCR-16/20 again showed two partially resolved peaks corresponding to monomer and dimer. The monomer showed an *s_20,w_* value of 2.8–3.1 S for 0.5–2 mg/ml while the dimer showed an *s_20,w_* value of 4.2 S. The percentage of dimer increased with concentration from 11% to 30%, leading to a *K_D_* value for dimer formation of 90 ± 20 µM (**Table 1**).
(iv) SCR-16/18H showed a monomer peak in the *c(s)* plot with an *s_20,w_* value of 2.2 S and a dimer peak with an *s_20,w_* value of 3.3–3.5 S. Monomer and dimer comprised 50% each, and the *K_D_* value for dimer formation was of the order 10 µM.
(v) SCR-17/18H showed a smaller monomer *c(s)* peak at a *s_20,w_* value of 2.2 S and a larger dimer peak at 3.5–3.8 S. The percentage of dimer was 80% and this corresponded to a *K_D_* value for dimer formation of 2 ± 1 µM.
(vi) SCR-18H exhibited a larger monomer *c(s)* peak at an *s_20,w_* value of 2.3 S and a smaller dimer peak at an *s_20,w_* value of 3.7 S. As for the 137 mM NaCl *c(s)* analysis, the *s_20, w_* and mass values were larger than expected for a 10 kDa protein. The percentage of monomer was 60% and the *K_D_* value for dimer formation was of the order of 60 µM.
(vii) SCR-17H showed a smaller monomer *c(s)* peak at an *s_20,w_* value of 1.5–1.8 S, and a larger dimer peak at an *s_20,w_* value of 2.5–2.8 S, with 80% dimer. The *K_D_* value for SCR-17H dimer formation was calculated to be 5 ± 3 µM. The *s_20,w_* and mass values were larger than expected for a 10 kDa protein.

Overall, the *c(s)* results for 50 mM NaCl buffer agreed with the *c(s)* results from the 137 mM NaCl buffer study above. The *K_D_* values were similar in 50 mM NaCl buffer when compared to those for 137 mM NaCl buffer. Importantly, it was deduced that electrostatic interactions were not significant in CFH dimer formation. SCR-17H was again identified as the dimer site because the three smaller fragments with this SCR-17 domain showed the lowest (strongest binding) *K_D_* values in a range of 2-10 µM. In contrast the three other *K_D_* values were 60, 90 and 180 µM.

### SAXS Results for the Seven CFH Fragments in 137 mM NaCl

**Figure 5:**
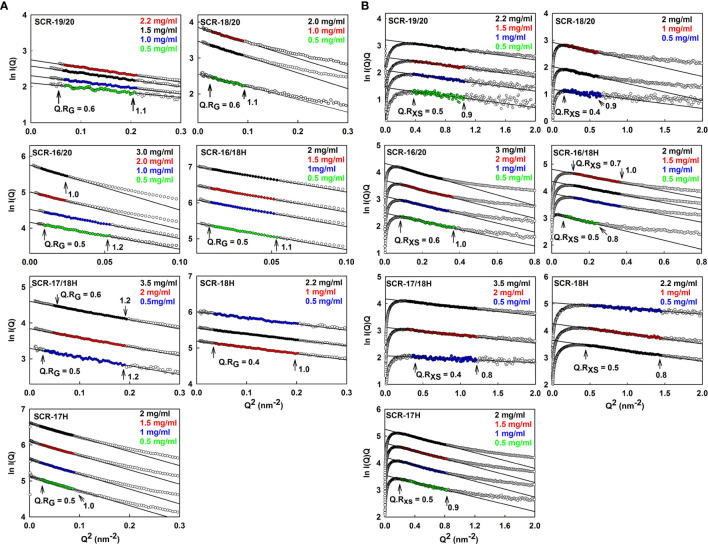
Guinier *R_G_* and *R_XS_* analyses for each of the seven SCR fragments. The filled circles represent the experimental X-ray data points used to determine the *R_G_* and *R_XS_* values. Their values were measured within the satisfactory *Q.R_G_* and *Q.R_XS_* ranges shown in each panel. **(A)** The SCR fragment concentrations in 137 mM NaCl buffer are shown in the panels, where the colors correspond to the indicated concentrations. The filled circles correspond to the *I(Q)* values used to determine each *R_G_* value. The *Q* ranges used for the *R_G_* fits were 0.24–0.44 nm^−1^ for SCR-19/20, 0.14–0.3 nm^−1^ for SCR-18/20, 0.10–0.22 nm^−1^ for SCR-16/20 monomer and SCR-16/18H, 0.10–0.14 nm^−1^ for the SCR-16/20 dimer, 0.20–0.44 nm^−1^ for SCR-17/18H, 0.17–0.45 nm^−1^ for SCR-18H and 0.14–0.28 nm^−1^ for SCR-17H. **(B)** The corresponding *R_XS_* analyses for the seven SCR fragments are shown, where the *Q* ranges used for the *R_XS_* fits were 0.55–1.02 nm^−1^ for SCR-19/20, 0.39–0.79 nm^−1^ for SCR-18/20, 0.28–0.6 nm^−1^ for the SCR-16/20 monomer, 0.28–0.50 nm^-1^ for the SCR-16/20 dimer, 0.22–0.49 nm^-1^ for the SCR-16/18H monomer, 0.35–0.60 nm^-1^ for the SCR-16/18H dimer, 0.53–1.09 nm^−1^ for SCR-17/18H, 0.45–0.88 nm^−1^ for SCR-18H and 0.7–1.19 nm^−1^ for SCR-17H.

SAXS yields size and shape information on macromolecules in solution (40). SAXS data with good signal to noise was obtained for the seven SCR fragments in 137 mM NaCl buffer. Guinier analysis were carried out on the subtracted curves to calculate the radius of gyration *R_G_* which is a measure of the overall elongation of the molecule (**Figure 5A**) and the *R_G_* of the cross section (*R_XS_*) (**Figure 5B**). Successful linear Guinier analyses for each of the SCR fragments were carried out within satisfactory fit limits of the *Q.R_G_* and *Q.R_XS_* values, namely 0.6–1.2 and 0.4–1.0 respectively. The *R_G_/R_O_*ratio compares the elongation of the protein with respect to a sphere, where *R_O_* is the *R_G_* of a perfect sphere with the same volume as the hydrated protein. Typical globular proteins have a *R_G_/R_O_* ratio of 1.28 (41). Solution scattering represents an average of the species present in the sample, and monomer and dimer could not be distinguished as such. Nonetheless scattering provides an independent monitor of the extent of SCR dimerization to complement the AUC data.

**Figure 6:**
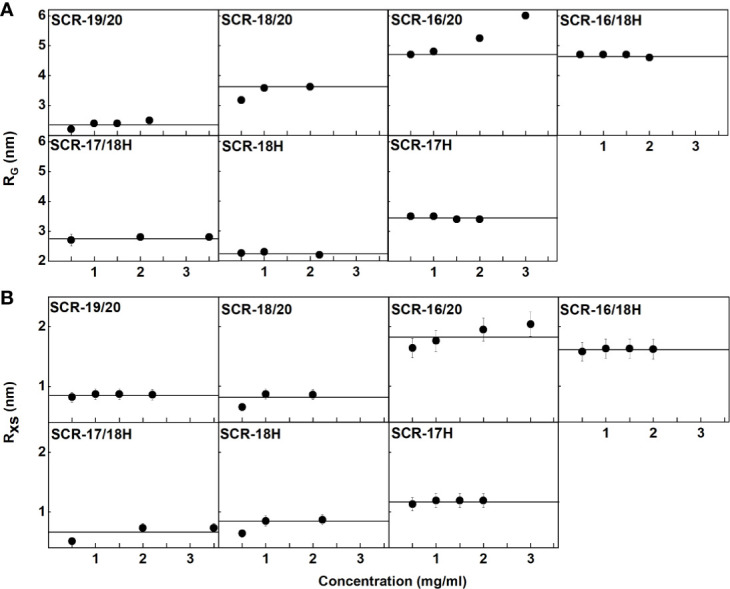
Concentration-dependence of the *R_G_* and *R_XS_* value of the seven SCR fragments. **(A)** The X-ray *R_G_* values are shown, where the lines denote the mean value. **(B)** The X-ray *R_XS_* values are shown, where the lines denote the mean value.

For the three non-His-tagged fragments SCR-19/20, SCR-18/20, and SCR-16/20, Guinier analyses were carried out:

(i) For SCR-19/20, the *R_G_* value was 2.4 ± 0.1 nm and the *R_G_/R_O_* ratio was 1.7 indicating that it was elongated with respect to a globular protein of the same size. SCR-19/20 had an *R_XS_* value of 0.86 ± 0.04 nm. Neither the *R_G_* nor the *R_XS_* changed significantly with respect to concentration as expected for the monomeric SCR-19/20 fragment (**Figures 6A, B**).
(ii) For SCR-18/20 the *R_G_* value increased with concentration from 3.2 ± 0.1 nm to 3.6 ± 0.03 nm (**Figure 6A**). The *R_G_/R_O_* ratio increased from 2.0 to 2.2 which showed that SCR-18/20 had an elongated shape which became further elongated upon dimer formation. The *R_XS_* value for SCR-18/20 increased from 0.9 ± 0.04 nm to 1.2 ± 0.1 nm (**Figure 6B**).
(iii) For SCR-16/20, the *R_G_* value increased from 4.7 ± 0.1 nm to 6.0 ± 0.1 nm (**Figure 6A**). The *R_G_/R_O_* ratio increased from 2.4 to 3.1, with SCR-16/20 becoming more elongated upon dimer formation. The *R_XS_* value increased with concentration from 1.6 ± 0.08 nm to 2.04 ± 0.1 nm (**Figure 6B**); the increase in *R_XS_* compared to that of SCR-19/20 showed that dimerization occurred by a side-to-side association of the five SCR domains.

For the four His-tagged SCR fragments, Guinier analyses showed that the *R_G_* values did not change significantly with concentration (**Figures 6A, B**).

(iv) SCR-16/18H had an *R_G_* value of 4.7 ± 0.1 nm and an *R_G_/R_O_* ratio of 2.7. The *R_XS_* value for SCR-16/18H was 1.6 ± 0.08 nm. These values resembled those for SCR-16/20.
(v) SCR-17/18H had an *R_G_* value of 2.8 ± 0.1 nm and an *R_G_/R_O_* ratio of 1.7. SCR-17/18H had an *R_XS_* value which increased at higher concentrations from 0.51 ± 0.03 nm to 0.73 ± 0.04 nm (**Figure 6B**). Its *R_G_* value was larger than that of SCR-19/20, this being attributed to its larger size and glycosylation.
(vi) SCR-18H had an *R_G_* value of 2.3 ± 0.03 nm, an *R_G_/R_O_* ratio of 1.4, and an *R_XS_* value of 2.3 ± 0.1 nm. The similarity of its *R_G_* value to that of SCR-19/20 is consistent with SCR-18H existing as a mixture of monomer and dimer, although its glycosylation and His-tag will complicate this interpretation.
(vii) SCR-17H had an *R_G_* value of 3.5 ± 0.02 nm, an *R_G_/R_O_* ratio of 2.2, and an *R_XS_* value of 3.5 ± 0.2 nm. Its significantly larger *R_G_* and *R_XS_* values compared to SCR-19/20 and SCR-17/18H is attributed to its high level of dimer formation as well as its glycosylation and His-tag. This agrees with the 80% dimer seen in the AUC *c(s)* analyses.

**Figure 7:**
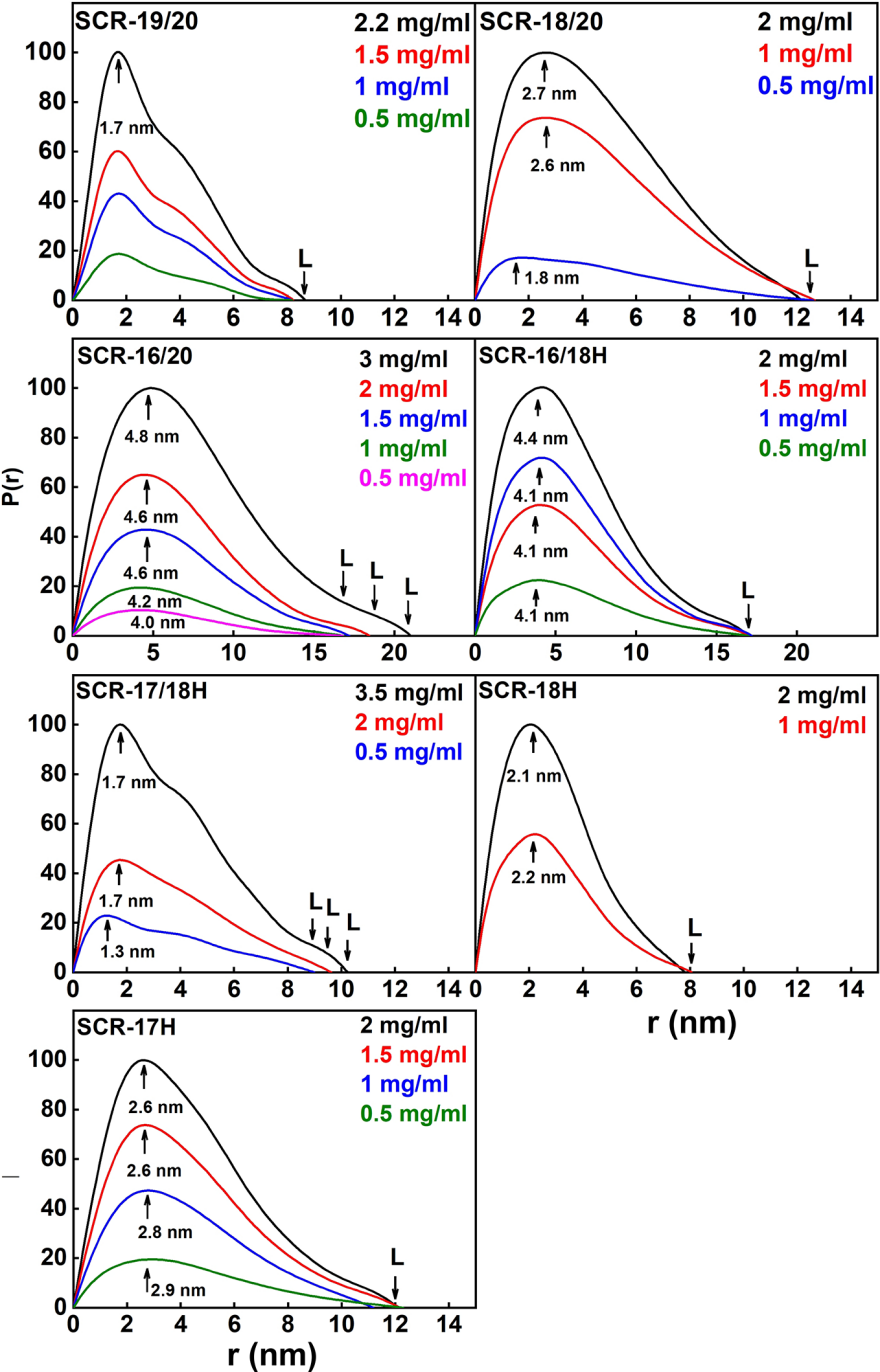
Distance distribution function *P(r)* analyses for each of the seven SCR fragments. The arrow under each peak represents M, the most frequent distance within the protein, and L represents the maximum observed dimension of the SCR fragment.

The distance distribution function *P(r)* provides the maximum dimension of the macromolecule L and the most frequently observed interatomic distance M (**Figure 7**; **Table 1**). As a check, the *P(r)* analyses were found to give *R_G_* values that agreed well with the Guinier *R_G_* values.

(i) SCR-19/20 gave a length L of 8.2 ± 0.4 nm and an M value of 1.7 ± 0.1 nm, neither of which changed with concentration as expected for a monomer.
(ii) SCR-18/20 gave a length L of 12.2 ± 0.6 nm and an M value of 2.7 ± 0.1 nm at high concentration. The increase in L is as expected from the addition of an extra SCR domain of length 3.6 nm to SCR-19/20.
(iii) For SCR-16/20, the length L increased with concentration from 16.8 ± 0.8 nm to 21 ± 1 nm. The M values also increased from 4.0 ± 0.2 nm to 4.8 ± 0.2 nm. This indicated increases in its dimerization with concentration, this being consistent with the AUC data.
(iv) SCR-16/18H gave a length L of 17 ± 0.8 nm and an M value of 4.1 ± 0.2 nm with no observed concentration dependent changes. The additional length when compared to SCR-18/20 indicated that the His-tag is extended in its conformation.
(v) For SCR-17/18H, its length L was between 9 ± 0.5 nm to 10.2 ± 0.5 nm with M values of 1.7 ± 0.1 nm. Because these lengths are not much greater than that for SCR-19/20, this outcome suggested that dimerization occurred through a side-by-side association of SCR-17/18.
(vi) SCR-18H had a length of 8 ± 0.4 nm and an M value of 2.1 ± 0.1 nm. These relatively large values are consistent with dimer formation and the presence of glycosylation and the His-tag in its structure.
(vii) SCR-17H had an L value of 12 ± 0.6 nm with an M value of 2.6 ± 0.1 nm. These larger values compared to SCR-18H showed that more dimer was present in this case, as suggested by the AUC analyses.

Overall the SAXS analyses confirmed the AUC analyses that showed that SCR-19/20 was monomeric, and that dimerization occurred for the other six SCR fragments. The *R_XS_* and L values suggested that dimer formation occurred as a side-by-side association and not as an end-to-end association.

### SAXS Results for the Seven CFH Fragments in 50 mM NaCl

Similar SAXS analyses were performed on each of the seven SCR fragments in buffers containing 50 mM NaCl as a control to check the effect of low salt on protein dimerization (data not shown; **Table 1**) (39). The Guinier *R_G_* and *R_XS_* plots gave high quality linear fits, as exemplified in **Figure 5** for 137 mM NaCl buffer, within the satisfactory fit limits of the *Q.R_G_* and *Q.R_XS_* values as above. For the seven SCR fragments, similar *R_G_* and *R_XS_* results to those in 137 mM NaCl were obtained (**Table 1**). The distance distribution *P(r)* curves for each of the SCR domains were likewise calculated for comparison with those obtained for 137 mM NaCl (not shown). Again, little difference in the lengths L was observed between 137 mM and 50 mM NaCl (**Table 1**). As for the AUC results, lowering the ionic strength of the buffer did not significantly alter the calculated SAXS parameters for the SCR fragments. This confirmed the above AUC results that electrostatic interactions were not significant in dimer formation.

### Modelling of the SAXS Curves for the Seven CFH Fragments

Atomistic scattering modelling reproduces the SAXS scattering curves of a macromolecule by recourse to physically-realistic molecular models created from Monte Carlo and molecular dynamics simulations (42). To model the seven C-terminal SCR fragments in this study, we used the recently published scattering model of full-length CFH to create molecular structures for the seven individual CFH fragments (6). That study generated 29,715 physically-realistic conformationally-randomized structures for CFH, from which the 100 best-fit structures to the scattering curve of full-length CFH were identified, as well as a single best-fit median CFH structure. Starting from the best fit model of that study, the seven SCR fragments were created by edits of the full-length CFH structure. The C-terminal His-tags were modelled onto four of these fragments (**Supplementary Figure 1**). For the three non-His-tagged models, additional molecular structures for these fragments were extracted from the 100 best-fit CFH structures in order to assess the variability of the calculations between different structures in that set.

**Figure 8:**
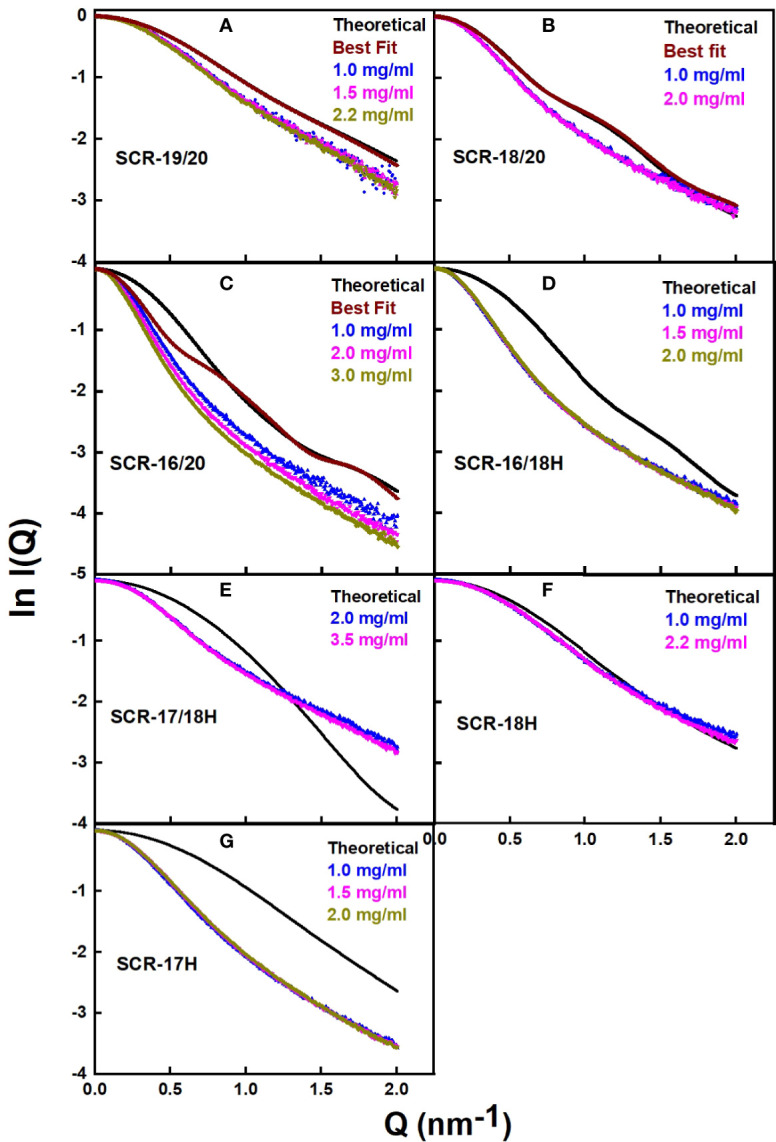
Scattering curve fits for the atomistic models for each of the SCR fragments. **(A–G)** For each of the seven SCR fragments, the panels compare the experimental X-ray scattering and theoretical scattering curves. The theoretical curves are shown in black, and the experimental curves are shown in color to correspond to the different concentrations shown in the same panel. In **(A–C)**, curves from the 100 best-fit models are shown in brown.

The scattering curve fits (**Figure 8**) confirmed the above AUC and SAXS results on the dimerization of the CFH fragments. Because the SAXS data represent an average of the species present in solution, the fits do not distinguish monomer and dimer, but rather the deviation of the fits from an assumed monomer structure. The modelling did not consider directly the occurrence of dimer formation because the molecular structures of the dimers were unknown. However, by calculating the goodness-of-fit R-factor for each fragment model when compared with up to three different experimental curves, and by assuming that the monomer models would give good curve fits if the solution structure was monomeric, it was possible to assess the extent to which dimerization had perturbed the curve fits. As a bench mark, SCR-19/20 should give the best fits as this was determined to be monomeric by AUC and SAXS. The reasonable visual fits (**Figure 8A**) and R-factors of 14–17% (**Table 2**) indicated the type of fits that were obtained Relatively low amounts of dimer were seen for SCR-18/20 by AUC (**Figure 1B**) and SAXS; the visual fits (**Figure 8B**) and R-factors of 15% (**Table 2**) corroborated the relatively low amounts of dimer present for this fragment. For SCR-16/20, the presence of 40% dimers by AUC were confirmed by increases in the R-factors from 40 up to 64% as the concentration (and proportion of dimer) increased. For the four His-tagged fragments, while SCR-18H showed good agreements in the curve fits, the fits for SCR-16/18H, SCR-17/18H, and SCR-17H were poorer and gave R-factors as high as 68%. Taken together, the R-factors indicated that the primary location of CFH dimerization was again seen to be SCR-17, with some contribution from SCR-18.

**Table 2:** The modelling of the AUC $s_{20,w}^{0}$ values and the best curve fit R-factors for the SCR fragments.

Fragment	$s_{20,w}^{0}$ (S) experimental	$s_{20,w}^{0}$ (S) modelled	R-factor 1.0 mg/ml	1.5 mg/ml	2.0 mg/ml	3.5 mg/ml
SCR-19/20	1.4	1.4	14.4%	15.0%	17.1%	
SCR-18/20	2.4	1.9	14.5%		15.2%	
SCR-16/20	2.7	2.7	40.2%		53.9%	64.4%
SCR-16/18H	2.3	1.9	51.2%	51.0%	49.5%	
SCR-17/18H	2.3	1.8			22.5%	23.0%
SCR-18H	2.4	1.1	6.4%		5.8%	
SCR-17H	1.7	1.2	68.0%	66.0%	65.2%	

The R-factor was calculated using the R-factor metric in the SASSIE package. For each SCR fragment, the R-factor was calculated at each of the concentrations in use from 1.0 mg/ml to 3.5 mg/ml.

**Figure 9:**
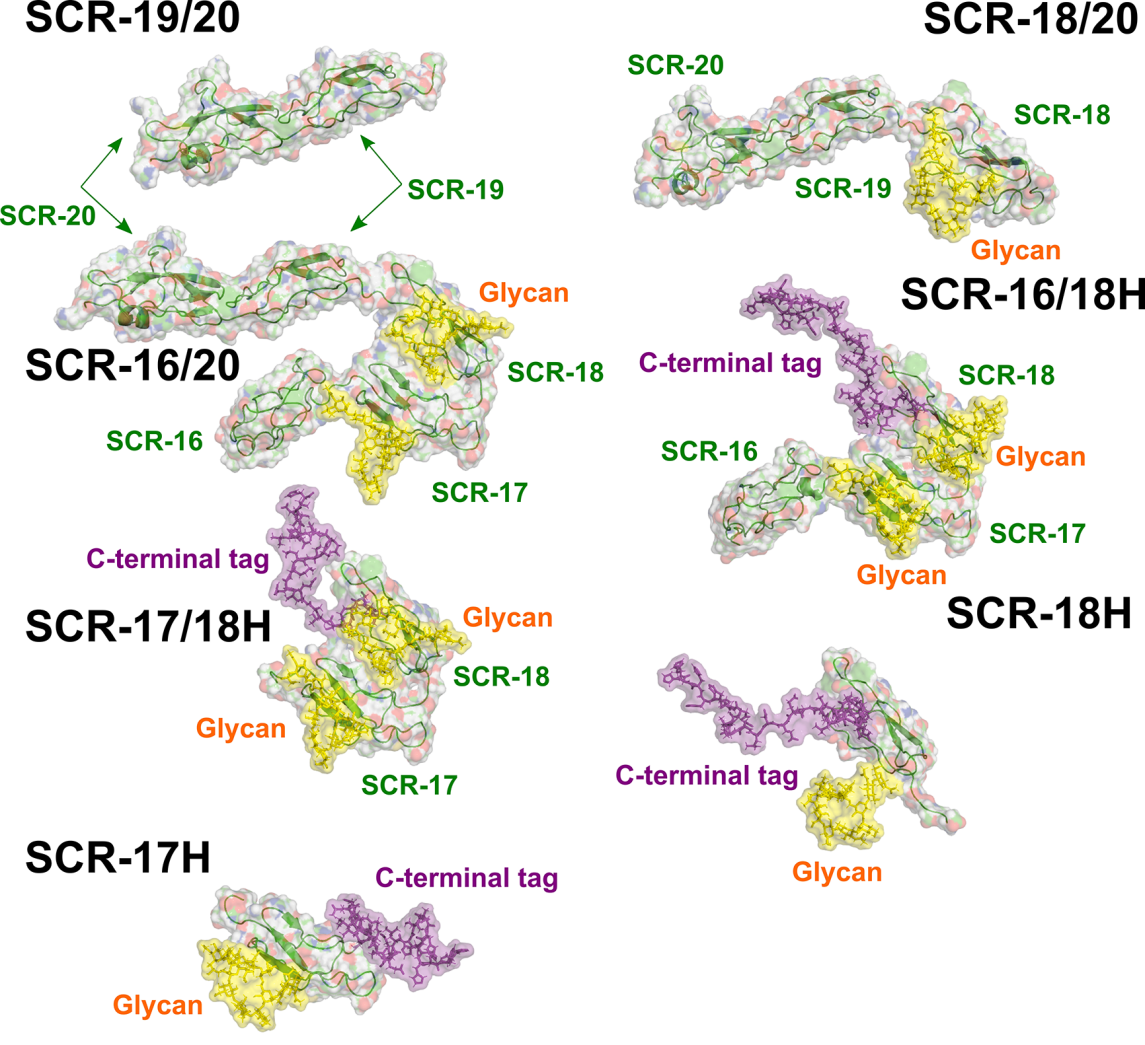
The seven atomistic models for the SCR fragments. The structures of the C-terminal fragments of CFH used for the curve fits are shown. The individual SCR domains are labelled. Yellow surfaces represent the single glycan chains located on each of SCR-17 and SCR-18. The C-terminal tag is shown as a purple surface.

As another test of the scattering modelling, the $s_{20,w}^{0}$ values for the models of the seven monomeric CFH fragments (**Figure 9**) were calculated using HYDROPRO (**Table 2**). Given that the mean difference between the modelled and experimental values should be ± 0.21 S for related macromolecules (43), excellent agreements were obtained for monomeric SCR-19/20 and SCR-16/20, but less so for the SCR-18/20 monomer where the model appeared too elongated compared to the experimental value. For the four His-tagged fragments in **Table 2**, the calculations suggested that the models were too elongated compared to the experimental values. The simplest explanation of this is that the His-tag tails and glycans were extended in the models as shown in **Figure 9**. While these calculations corroborated the modelling for the monomeric fragments, overall the agreement was only qualitative.

Further experimental AUC analyses were made using the *f/f_0_* ratio which monitors the extent of how much the frictional coefficient of the glycoprotein deviates from that for a sphere of the same volume. The experimental *f/f_0_* ratio of 1.1 for monomeric SCR-19/20 defined a benchmark for two linearly-arranged SCR domains (**Table 1**; **Figure 9**). SCR-18/20 also showed an experimentally low *f/f_0_* ratio of 1.1 (**Table 1**). As deduced from the *f/f_0_* ratios of 1.3–1.5 (**Table 1**), the three SCR-16/20, SCR-16/18H, and SCR-17/18H monomers have similar but more elongated shapes. The similarity of the *f/f_0_* ratios of 1.3 for SCR-16/18H and SCR-17/18H indicated that the three-domain SCR-16/18H structure possessed a bent back solution structure of similar elongation to the two-domain SCR-17/18H structure. The sedimentation properties of the single SCR-17H and SCR-18H domains are likely to be perturbed by their relatively large glycan and His-tag groups, thus no further interpretation of their values was made here. It was however interesting that the *f/f_0_* ratios for the dimers of SCR-16/20, SCR-18/20, SCR-16/18H, SCR-17H, and SCR-18H showed that they became more elongated in their dimers compared to their monomers (**Table 1**). However, that for SCR-17/18H was unchanged in its dimer, suggesting that this was formed by a side-by-side interaction.

Previously SCR dimers have been seen in the crystal structure of FHR1 SCR-1/2, in which two copies of SCR-1/2 formed an anti-parallel dimer. This anti-parallel dimer structure was used to test whether the FH SCR-17/18 dimer could be formed from protein-protein contacts in the same way as the dimer interface seen in the FHR1 SCR-1/2 structure. Using the crystal structure of FHR1 as a template (PDB code 3ZD2), a model of the SCR17/18 dimer interface was constructed. A multiple sequence alignment between FHR1-SCR1/2 and SCR-17/18 of CFH gave a relatively low sequence homology of 41% between the two fragments. When CFH SCR-17 was aligned with FHR1 SCR-1, and SCR-18 was aligned with SCR-2 through their β4-strands, the resulting SCR-17/18 dimer model showed ill-fitting gaps at their interface. These observations argued against an antiparallel SCR arrangement for the C-terminal CFH dimer at SCR-17 and SCR-18. Accordingly, because each of SCR-17H and SCR-18H form dimers on their own (**Figure 4B**), it was concluded that a parallel arrangement of the SCR-17/18 domains is found in the SCR-17/18 dimer detected by AUC analysis.

## Discussion

Full length CFH forms weak dimers with an estimated range of 4–15% dimer present at typical CFH serum concentrations of 0.8–3.6 µM (0.116–0.562 mg/ml) (44). The dissociation constants *K_D_* values for dimer formation ranged between 8–28 µM (45), thus CFH dimers are expected to co-exist with CFH monomers at physiological conditions in serum. The CFH self-association sites have previously been shown to be located in the SCR-6/8 and SCR-16/20 regions (12, 13, 46). Up to now, the more precise location of the SCR-16/20 dimerization site was not known, and this identification was addressed here. It was unlikely that the dimerization site would reside on either SCR-19 or SCR-20, because of the functional interaction of SCR-19 and SCR-20 with the C3d fragment of complement C3b and sialic acid as reported in crystal structures (47–51) (PDB codes 3OXU, 2XQW, 4ONT, 4ZH1, 5NBQ). This was confirmed in this study by showing that SCR-19/20 remained monomeric using a combination of AUC and SAXS experiments in 137 mM and 50 mM NaCl buffers, coupled with molecular simulations of the AUC and SAXS data based on the coordinates of our recent full-length CFH model (6). In contrast, using the same strategy, six other fragments containing SCR-17 and SCR-18 showed various degrees of dimer formation. The strongest dimer formation with *K_D_* values of 3-6 µM was observed for SCR-17H, SCR-17/18H, and SCR-16/18H, in which SCR-17H showed higher dimer formation than SCR-18H. These data indicated that SCR-17H comprised the main C-terminal CFH dimer site. The presence of additional or alternative SCR domains as found in the SCR-16/20, SCR-18/20H, and SCR-18H fragments resulted in weaker dimer formation, indicating that SCR-18H made some contribution to this. Since full-length CFH showed extents of dimerization of 4–15% (12), while the smallest fragments showed dimerization of up to 80% (**Figure 4B**), the reduced dimer formation for larger CFH molecules is attributed to steric effects caused by the larger sizes of the CFH proteins in question. Their larger sizes are presumed to inhibit dimer formation. It should also be noted that the present study used a non-His tagged form of SCR-16/20, while our previous study used a His-tagged variant (13). Both studies gave similar AUC results, indicating that the presence of the His-tag made no difference on its dimerization.

**Figure 10:**
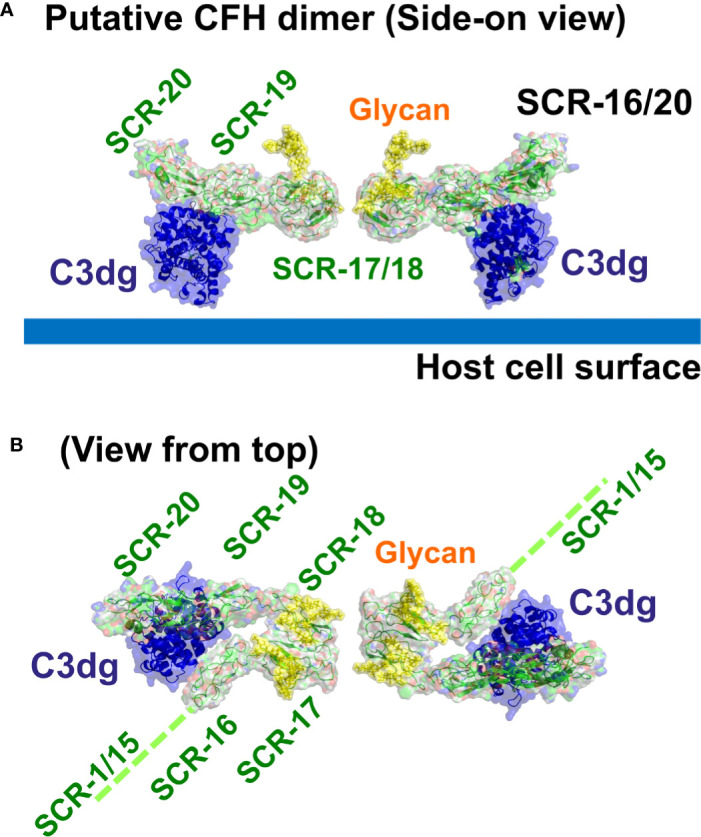
Putative dimer of SCR-16/20 to show the crystallographic-observed complex between SCR-19/20 and C3dg. Molecular views of the SCR-16/20 fragment model (grey/green) determined from this study are shown as ribbons and surfaces to show how this binds to its ligand C3dg (blue) on the SCR-19 domain. To generate this complex, the SCR-16/20 model was superimposed onto the crystal structure of the SCR-19/20 and C3dg co-complex (PDB code: 5NBQ). The yellow surfaces show the two glycan chains located at each of SCR-17 and SCR-18. **(A)** The side-on view shows C3dg attached to the host cell surface (thick blue line) through its thioester group, as well as showing how SCR-17 and SCR-18 form a dimeric interface with another SCR-16/20 molecule (**Figure 4B**). **(B)** The same structure is viewed from the top to show the five individual SCR domains in each monomer of the SCR-16/20 dimer. This view corresponds to a 90° rotation about a horizontal axis compared to that in **(A)** The reminder of the CFH structure SCR-1/15 is denoted by a dashed green line.

The major complement regulator CFH functions to protect host cells from destruction through its C-terminal binding to C3b and anionic host cell surfaces mediated by the SCR-19 and SCR-20 domains. Our novel report of a CFH dimer site in SCR-17/18 may provide CFH with a functional mechanism through which CFH can become more concentrated on host surfaces during an inflammatory response. The SCR-17/18 dimer site is seen to be independent of the crystallographic-observed C3dg and anionic oligosaccharide binding sites on SCR-19/20 in the C-terminal region of CFH (**Figure 10**). Despite the presence of multiple binding sites for C3dg and polyanions, the schematic view of C3dg binding to SCR-19 and the dimer formation at SCR-17/18 in **Figure 10** indicated that dimer formation will still proceed when a single CFH molecule is bound to a host cell surface through either cell-bound C3dg or polyanions. The single surface-bound CFH molecule will allow an additional CFH molecule to be recruited through a SCR-17/18 dimerization event to protect further the host surface under inflammatory conditions of excessive C3b deposition. In reflection of this topology, the 37 genetic variants throughout SCR-20 reported so far that lead to aHUS disease are comparatively abundant in CFH (**Figures 1B, C**) (17), indicating the importance of C-terminal CFH binding to host cell surfaces. The fewer variants reported to date for SCR-16 (six), SCR-17 (four), SCR-18 (ten) and SCR-19 (seven) might involve incorrect folding of the C-terminal domains if they alter any of the highly conserved Cys or Trp residues, or a reduction in dimer formation or C3dg binding (19). Visual inspection of the locations of these variants in the SCR-16/20 model (**Figures 1B, 10**) showed little further insight. Further experimental studies will identify the effect of the aHUS variants in SCR-17/18 on dimer formation in order to clarify the importance of C-terminal dimerization in CFH. Of interest was that no significant differences in dimer formation were observed between the 137 mM and 50 mM NaCl buffers, suggesting that electrostatic interactions were not significant in SCR-16/20 dimer formation (**Table 1**).

Besides CFH itself, another distinct SCR dimer has been observed in other members of the CFH gene family. The FHR proteins (52) including FHR1 with five SCR domains, FHR2 with four SCR domains, and FHR5 with nine SCR domains exist as dimers formed by an antiparallel pairing of their SCR-1/2 N-terminal domains, as opposed to the parallel pairing proposed for SCR-17/18. FHR dimerization confers avidity for their binding to complement activation fragments bound to host cell surfaces, and enables these FHR proteins to compete with CFH for binding (36). For FHR5, we showed that the antiparallel FHR5 dimer with 18 SCR domains has a compact domain structure that can bind bivalently to C3b when this is bound to host cells at a high enough surface density (53). However, sequence alignments between SCR-1/2 of FHR1 and SCR-17/18 of CFH showed that the three FHR1 residues (Tyr 34, Ser 36, and Tyr 39) essential for FHR1 dimer formation were not conserved in SCR-17/18 which contains Thr residues at the equivalent positions. This difference makes it unlikely that SCR-1/2 of FHR1 would be a good model for the CFH dimer structure at SCR-17/18.

Our multidisciplinary approach to analyse the solution properties of the seven C-terminal SCR fragments showed consistent results from both the AUC and SAXS data sets. The main results showed that SCR-19/20 is monomeric, and that SCR-16/20 and SCR-18/20 became more elongated with dimer formation (**Table 1**). For the remaining fragments, the AUC data showed a range of dimer formation had taken place, although information from SAXS about shape or size changes associated with dimer formation was more limited because the scattering curves correspond to mixtures of monomer and dimer, and the AUC shape data were of limited precision. Nonetheless there was sufficient information in the datasets to indicate that SCR-17 and SCR-18 comprised the main dimerization site in the C-terminal region of CFH. Both the AUC and SAXS data sets were accessible to molecular modelling in order to clarify the significance of the experimental data sets. The application of modelling here confirmed that the largest deviations from the SAXS curve fits on the assumption of SCR monomers correlated with the greatest amount of dimer formation (**Table 2**), as well as showing that the CFH monomer models accounted well for the AUC *s_20,w_*values. In this analysis, the AUC and SAXS modelling outcomes extended our understanding of the proportions of monomer and dimer deduced from AUC (**Figure 4B**). The molecular modelling also provided a new functional explanation for the formation of SCR-17/18 dimers, and insight into why aHUS disease-associated genetic variants occur along the length of SCR-16/20 and not just in SCR-20 (**Figure 10**).

## Author’s Note

Initial data from this work were presented at the 15th European Meeting on Complement in Human Disease, September 8–12, 2015, Uppsala, Sweden (Mol. Immunol. 2015, 67:136-136).

## Data Availability Statement

Requests to access the datasets should be directed to SP, s.perkins@ucl.ac.uk.

## Author Contributions

SP initiated and supervised the study at UCL. OD, RN, and SP designed the experiments. PA and DG provided the *Pichia* expression systems. OD expressed and purified the recombinant proteins with advice and support from MM and MH. VF supervised the study in Grenoble. XG and SP performed the computational modelling. OD, XG, and SP wrote the manuscript with the help of the other authors. All authors contributed to the article and approved the submitted version.

## Funding

OD thanks University College London and the Institut-Laue-Langevin for a PhD studentship award. Support for this work was also provided in part by the CCP-SAS project, a joint EPSRC (EP/K039121/1) and NSF (CHE-1265821) grant.

## Conflict of Interest

The authors declare that the research was conducted in the absence of any commercial or financial relationships that could be construed as a potential conflict of interest.

## Abbreviations

C3, complement component 3; CFH, Factor H; SCR, short complement regulator.
