# Supplementary material for: A Dimerization Site at SCR-17/18 in Factor H Clarifies a New Mechanism for Complement Regulatory Control
Source: Front Immunol. 2021 Jan 21;11:601895. doi: 10.3389/fimmu.2020.601895 (PMC7859452; doi:10.3389/fimmu.2020.601895)
Supplement: Supplementary file 1 [file Table_1.docx]

**SCR-16 SCR-17 SCR-18 SCR-19 SCR-20**

**SCR-16/20** **EFGLP**CKSP.....PSC**IKTD**CLSL..ASNVTC..PTC**RDTS**CVNP..GNWTEP..PQC**KDSTGK**CGPP..PKC**LHPC**..PTC

**SCR-18/20 RDTS**CVNP..GNWTEP..PQC**KDSTGK**CGPP..PKC**LHP**C..PTC

**SCR-19/20** **EFSTGK**CGPP..PKC**LHP**C..PTC

**SCR-16/18H** **EFGLP**CKSP.....PSC**IKTD**CLSL..ASNVTC..PTC**RDTS**CVNP..GNWTEP..PQC_HISTAG

**SCR-17/18H** **IKTD**CLSL..ASNVTC..PTC**RDTS**CVNP..GNWTEP..PQC_HISTAG

**SCR-17H** **IKTD**CLSL..ASNVTC..PTC_HISTAG

**SCR-18H RDTS**CVNP..GNWTEP..PQC_HISTAG

**Histidine-tag C-terminal** PLEQKLISEEDLNSAVDHHHHHH

**SUPPLEMENTARY FIGURE 1 |** The sequences of each of the modelled SCR fragments based on the SCR-16 to SCR-20 domain.

Highlighted in grey are the four conserved Cys residues. The inter-SCR linker regions for each SCR domain are highlighted in bold. For SCR domains expressed with a hexa-histidine tag, namely SCR-16/18H, SCR-17/18H, SCR-17H and SCR-18H, the C-terminal tags are denoted as HISTAG. The full His-tag C-terminal sequence is listed at the bottom.
